# Auditory-Verbal Processing Disorder and Dyslexia in Adulthood

**Published:** 2019-01-12

**Authors:** C Cassandro, A Manassero, A Scarpa, V Landi, G Aschero, S Lovallo, P Velardo, P De Luca, A Albera, R Albera, E Cassandro

**Affiliations:** 1 Surgical Sciences Department, University of Turin, Turin, Italy; 2 City of Health and Science, University Hospital of Turin, Italy; 3 Department of Medicine and Surgery, University of Salerno, Salerno, Italy

## Introduction

According to the Diagnostic and Statistic Manual of Mental Disorders (DSM-V), the neurodevelopment disorders are characterised by an atypical development of some cognitive domains, with a dysfunctional behavioural correlation supported by phenotypes with a genetic connection.

Among these are the specific learning disabilities, which are united by a single condition characterised by difficulty in learning and using academic skills in the presence of an adequate IQ; these include dyslexia, dyscalculia and dysorthography.

The clinical features are an inaccurate or slow and laborious reading of the words, difficulty in understanding the meaning of what is read, difficulty in spelling, in written expression, in mastering the concept of number, numerical data ora calculation, and in arithmetic reasoning.

Recently, thanks to the better understanding of the neurobiological mechanisms underlying these disorders and thanks to the improvement of neuroimaging and genetic analysis, we better understood the evolution of these pathologies and consequently we have refined the diagnosis and treatment.

Studies focused particularly more on dyslexia, both due to its high frequency in general population and to the better understanding of its pathogenesis.

Various cognitive models are developed to explain the nature of the processes underlying the recognition of written words; the models explained partially the cause of dyslexia, tracing it back to a phonological deficit of consciousness. Today none of these models seems to prevail, but we believe that, in the context of a general reading disorder, there are several difficulties that can be either phonological or about phonological, motor or visual consciousness, due to the failure of magnocellular/auditory neurons development, because of an altered central processing.

Already in the 80s, Tallal et al. recognised the phonological deficit as a symptom of a deficit of sound processing presented in rapid sequence (proper of verbal oral language) and therefore a deficit of elaboration at the auditory temporal cortex. This difficulty in the sounds analysis could lead to a wrong categorization and to a failure to recognise the phoneme as the acoustic characteristics change.

From studies carried out on children with dyslexia, deficient performances emerged in conditions of short auditory stimuli presented in rapid sequence, while the performance improved with a longer stimulus interval. These difficulties lead to a need for longer times to discriminate short sounds in rapid sequence. Tallal deduced that children with dyslexia presented not a linguistic deficit but a deficit of temporal elaboration of the variation of the auditory patterns, which then translated into a difficulty in the perception of isolated language.

In auditory perception of sounds, both when proposed in rapid sequences and with a longer time intervals, a primary role is played by auditory attention, expecially by focused auditory attention.

The aim of this work is to present the features of adult patients wit a specific learning disability in reading and to evaluate the possible correlation with phonological awareness deficits (therefore the ability to analyse and manipulate the linguistic structure of words), and with auditory-verbal processing disorders.

## Materials and Methods

The study sample consisted of 70 patient (30 males and 40 females) aged between 17 and 55 years. 40% of the subjects were in the 18–19 age group; 36% between 20 and 25 years, while the remaining 24% were over 25 years old.

Of the 70 patients, 55% studied more than 13 years, 44% studied between 12 and 13 years, 3% studied only 10 years.

All patients approached an Audiology Unit to assess their learning skills.

The patients included in the study had the following requirements:

1. IQ Assesment
  DMS-V considers an IQ below the norm (<70 points) as an exclusion criteria. The subjects carried out a cognitive level assessment that highlighted the are a with best functioning and those that result as a weakness. In particular, the WAIS-IV (Wechsler Adult Intelligence Scale - Forth Edition) multi-behavioral test detects four dimensions; namely verbal comprehension, visual-preceptive reasoning, working memory, processing speed.
2. Absence of neurological and sensory deficits
3. Instrumental evaluation
  The protocol included some test: ADCL; Raven PM38; reading, comprehension, passage, writing calculation test; Boston Naming Test; lacking in phonemic, semantic influence and rapid naming tests of letters, figures and colours; barrage of figures; Night & Day Test; orientation of Benton lines; counting backwards; Beck’s inventory of depression-2; Hamilton Anxiety Rating Scale.
4. Positive screening of phonological consciousness
  Repetiton tests with scores lower than 16/20, especially when confirmed by unshielded mouth tests, require further evaluation of the auditory perceptive system.
5. Pure Tone Audiometry, Vocal Audiometry, Matrix Test
  The Matrix Test evaluates the average noise discrimination threshold (the stimulus/noise ratio), relative to the 50% of intellegibility. The reference value for nomoacusia is −6.7 +/− 0.7 dB (average +/− standard deviation). Base on the characteristics of the patients examinated, the average discrimination threshold was raised, to −3.8 dB.

## Results

Among 70 patients examined, 33% presented an insufficient noise threshold (SRT); of these, 56% showed a low repetition of non-words with shielded mouth, 61% a speed less than 4th percentage during the segmentation test, and 39% less than the 5th percentage during the fusion test.

After that we evaluated the patients with low SRT through some neuropsychological tests, as the piece dictation, the Night & Day Test and Milan calculation test; all these trials included a double task and the use of the working memory. During the dictation, 87% of the patients totalized a number of errors which places them under the 5th percentile; during the Night & day Test, only 61% of the subjects presented a speed performance above the 95th percentile.

During the calculation tests, 78% got a score below 2 ds. Evaluating the cognitive profile of patients with low SRT, 30% showed a medium-low IQ (>90) with homogeneous falls in three of four indexes examined (verbal comprehension index 26%; perceptual reasoning index 26%; processing speed index 22%). About the working memory, the deficit seemed to be greater (35%). Among the examined profiles, emerged 4 cases of sensorineural hearing impairment, 2 cases of conductive hearing loss, 4 cases of high auditory-verbal dissociation.

## Discussion

From the results obtained, we can hypothesise a comorbidity between the reading disorder and the auditory-verbal dissociation.

Through the analysis of the patient wit a loss auditory-verbal dissociation, we observed considerable difficulties in the repetition tests of non-words with shielded mouth, segmentation and fusion.

Then we examined how these patients answered to tests expecting a double task and the recruitment of the working memory (Dictation, Night & Day Test, Milan Test) we found an high percentage of low results understood as an high number of errors in the dictation and, in terms of speed, in attention and calculating test.

One third of patients with deficient SRT had a medium-low IQ (>90); this led us to endorse the multi componential theory of Evolutive.

The study didn’t analyse data from Benton Line Orientation Test (which could provide important information on visual analysis and processing matched with perceptive reasoning index of WAIS IV Test); a low score in this test should lead the examination to support the Magno-parvo-cellular theory.

Furthermore we didn’t carry out a qualitative evaluation of the written papers, in which phonological, non-phonological and perceptive-visual errors should confirm the Magno.parvo-cellular theory.

From the analysis of the whole sample, we found10 cases that could not be classified as mixed disturbance of scholastic capacity (4 cases of sensorineural hearing impairment, 2 of conductive hearing impairment, 4 case of high verbal auditory dissociation).

## Conclusion

The purpose of this study was to detect auditory-verbal alterations in patients belonging to our service for the diagnosis of ED, highlighting the correlation between the Matrix test and auditory perceptive phonological awareness screening.

We particularly focused on audiometric profile, phonological awareness, cognitive level and performance of instrumental skills (reading, writing, calculation).

Eve considering the importance of auditory processing of speech sounds, because it mimics a correct phonological representation and therefore a graphic relationship, we believe that the only perceptive-auditory alteration is not sufficient to cause a disorder such as evolutive dyslexia (ED).

We drawn up a table data based on the performance obtained from the Marix Test, pure tone audiometry, IQ evaluation, trial of the short-term memory test, quick naming test, linguistic tests such as the Boston Naming Test, test of physiological awareness, repetition tests and from the analysis of errors in writing tests.

The results of this work would seem to agree with the thesis that sees DE as a multifactorial disorder with possible phonological deficit with a possibile deficit of auditory perception.

Would be needed more studies with a a larger sample for further investigation.
